# Bio-Inspired Swarm Intelligence Optimization Algorithm-Aided Hybrid TDOA/AOA-Based Localization

**DOI:** 10.3390/biomimetics8020186

**Published:** 2023-04-29

**Authors:** Li Cao, Haishao Chen, Yaodan Chen, Yinggao Yue, Xin Zhang

**Affiliations:** 1 School of Intelligent Manufacturing and Electronic Engineering, Wenzhou University of Technology, Wenzhou 325035, China; 2 Key Laboratory of Intelligent Image Processing and Analysis, Wenzhou University, Wenzhou 325035, China

**Keywords:** hybrid localization, mobile location estimation, crow search algorithm, particle swarm optimization, maximum likelihood estimation

## Abstract

A TDOA/AOA hybrid location algorithm based on the crow search algorithm optimized by particle swarm optimization is proposed to address the challenge of solving the nonlinear equation of time of arrival (TDOA/AOA) location in the non-line-of-sight (NLoS) environment. This algorithm keeps its optimization mechanism on the basis of enhancing the performance of the original algorithm. To obtain a better fitness value throughout the optimization process and increase the algorithm’s optimization accuracy, the fitness function based on maximum likelihood estimation is modified. In order to speed up algorithm convergence and decrease needless global search without compromising population diversity, an initial solution is simultaneously added to the starting population location. Simulation findings demonstrate that the suggested method outperforms the TDOA/AOA algorithm and other comparable algorithms, including Taylor, Chan, PSO, CPSO, and basic CSA algorithms. The approach performs well in terms of robustness, convergence speed, and node positioning accuracy.

## 1. Introduction

With the advancement of wireless communication and navigation systems in recent years, wireless positioning technology has drawn increasing amounts of interest [1]. The Global Positioning System (GPS) is a mobile communication positioning technology that, according to the theory behind it, uses navigation satellites in orbit to measure time and distance in order to provide location information [2,3]. The GPS positioning system consists of three parts: a space satellite navigation part, a ground control part, and a user terminal part. There are now four major global positioning systems, including GPS from the United States, Beidou from China, GLONASS from Russia, and Galileo from the European Union, all of which are approved by the UN [4]. The GPS and Beidou systems are now the most dependable and stable of these four navigation systems. Currently [5,6], China has completed the layout of the Beidou satellite in terms of global coverage. In addition to offering precise location services, Beidou also includes satellite messaging and timing capabilities. China has now attained complete coverage, particularly in the military sector [7]. The Beidou system is also widely utilized in field operation communication, monitoring, and detection, as well as in the suppression of forest fires. The current location of the mobile station may be calculated using a specialized algorithm by evaluating several characteristics of the received radio waves [8,9].

For the wireless location methods investigated in mobile communications, there are two choices for implementation. Receivers (base stations), which measure the direction of arrival (angle of incidence of radio waves) of the received signal on the transmission path from the mobile station to more than two base stations through an array of antennas, use direction-based positioning techniques, such as angle of arrival (AOA: angle of arrival) positioning techniques, to determine their locations [10,11]. Distance-based positioning methods such as time difference of arrival (TDOA, time difference of arrival) positioning technology, which uses base stations to detect the time difference between the signal and the mobile station to transmit the signal, can be used to determine a hyperbola [12]. At least three base stations are required in order to locate the mobile station, although more are possible. Due to the faux-visible range (nondirect path) in indoor spaces, where the reflected or scattered signals can cause significant positioning errors, the current AOA positioning technology is not suitable for indoor positioning systems, but rather for suburban areas where the influence of multipath is small [13]. A slight variation in the base station’s placement angle when the mobile station is far from it will result in a significant mistake in the positioning distance and have an impact on positioning accuracy [14]. TDOA positioning science and technology, which is more effective in the error environment but also has a greater reliance on the time reference, requires full time synchronization between all the base stations involved in positioning, but does not need to know the time of transmitting from the mobile station, nor does it require synchronization between the mobile station and the base station. However, in the nonvisual range scenario, the positioning accuracy is low and prone to ambiguous answers, and the performance is considerably diminished [15].

Researchers developed a hybrid TDOA/AOA positioning algorithm as a solution to the aforementioned issues with AOA or TDOA [16]. The TOA-AOA positioning method combines the TOA and AOA positioning techniques. The fundamental tenet of this system is that the mobile station’s service base station monitors the angle and time at which its transmit signal approaches the mobile station [17]. The hybrid TDOA/AOA positioning algorithm only needs to synchronize the time of the stationary station and the time of the serving base station, which can be accomplished through the synchronization channel of the base stations. The emission signal should contain the emission time marker, just like the TOA localization method. To determine the location of the mobile station using the TOA-AOA positioning method, only one base station must be engaged in the measurement. When compared with either the TDOA or AOA algorithm alone, the TDOA/AOA hybrid localization method performs more accurately. The primary objective and difficulty of various TDOA/AOA hybrid positioning methods is how to solve the nonlinear formula system formed by these two different types of equations that are nonlinear. TDOA establishes the equation of MS position by the time difference of radio wave arrival and AOA establishes the equation of MS position by the angle of signal arrival [18].

The swarm intelligence optimization crow search algorithm (CSA) performs well when identifying high-dimensional functions and selecting features, among other things [19]. The technique is used for the first time in this study to solve the TDOA localization issue, and an enhancement strategy is put forth to provide a formula for computing the adaptive sensing probability. According to its model features, the passive time difference localization mathematical model is first examined and solved via crow search, and then the formula for adaptive sensing probability is created to adapt to the passive time difference localization issue. It overcomes the issue that other intelligent optimization algorithms tend to slip into local extremes and strikes a better balance between the global search ability and the local optimal search ability during the iterative phase. Finally, simulation findings demonstrate that the method works better than existing algorithms of the same kind and achieves good node localization accuracy, convergence speed, and robustness outcomes.

A TDOA/AOA hybrid location method built on the crow search algorithm and improved by particle swarm optimization is suggested to overcome the issues raised above. The particle swarm algorithm can converge rapidly and meet the goal of identifying the best. Before introducing the theory of chaos to the PSO algorithm and using the chaotic PSO algorithm to find the optimal value of the fitness function to obtain the optimal value of the estimated value of the mobile station, the algorithm first uses the maximum likelihood method to obtain the maximum likelihood estimation function of the mobile station and uses the maximum likelihood estimation function as the fitness function. The final simulation and comparative results demonstrate that the suggested approach performs better at localization.

The paper is organized as follows: Section 2 provides a discussion on the current progress of related research work, Section 3 describes in detail the TDOA/AOA hybrid location algorithm, Section 4 describes the crow search algorithm, and Section 5 provides a detailed introduction to the proposed CSA algorithm optimized by PSO algorithm, Section 6 describes in detail the application of the PSO-CSA algorithm to the TDOA/AOA location algorithm, Section 7 provides the algorithm comparison and result analysis. Section 8 provides the conclusions and directions for future works.

## 2. Related Work

The researchers incorporated other algorithms to the TDOA/AOA hybrid positioning algorithm to further refine it in light of its improved positioning performance in determining the location of the mobile station. First and foremost, much research has been conducted by current local and international academics to lessen the impact of measurement inaccuracies. For instance, Zhao et al. validated the Automatic dependent surveillance-broadcast (ADS-B) signal and to enhance positioning accuracy of enroute aircraft, an approach unite the technology of ADS-B and multilateration (MLAT) is presented, the hybrid technology improved the position accuracy [20].According to reference [21], a hybrid measurement approach is proposed to be used to create a new connection between unknown source coordinates, and the resultant mean square error matrix of the solution may be used to determine the CRB limit in the small error zone. The weighted least squares (WLS) technique, which was suggested in reference [22], is based on the first-order Taylor expansion of the noise factor and can lessen the estimate bias brought on by the least squares (LS) method. To further minimize the estimate bias when the target is beyond the sensor’s convex hull, a novel structured total least squares (STLS) approach is created. The precision of positioning is not only enhanced, but the benefit of low computational complexity is also maintained in reference [23], which addresses the issue of the traditional linear positioning algorithm’s poor positioning accuracy in an NLOS environment. According to reference [24], the quasi-normal distribution density curve should be used to create the TDOA error model in order to make it more accurate. In addition, the TOA measurement value is filtered using the Kalman filter to get it closer to the actual time of arrival, which is a real-time and direct way to remove mistakes. The least squares approach is enhanced and the new variables are split in the literature [25].

In recent years, several biological nature-inspired algorithms have been devised and inspired by natural events. These algorithms are extensively employed in TDOA/AOA hybrid localization algorithms to address their current issues [26]. This resolves the nonlinear optimization issue, and it is demonstrated that the bio-inspired algorithm performs better when the parameters are adjusted suitably in terms of convergence speed and accuracy. The TDOA localization problem was suggested to be solved using particle swarm optimization (PSO) in reference [27]. The method is highly accurate and does not require starting values for localization. An approach based on hybrid genetic quasi-Newton search was suggested in reference [28]. The algorithm lowers the later search rate and combines the benefits of the genetic algorithm and quasi-Newton algorithm. In an NLOS context, reference [29] developed a localization technique based on an ant colony optimization algorithm. Reference [30] solved the TDOA localization issue using the salp swarm algorithm (SSA), adopted a novel swarm updating model, and demonstrated the method’s efficacy in doing so. With its great stability and efficiency in handling issues such as nonlinear optimization and huge data processing, bio-inspired algorithms have emerged as a significant research area in artificial intelligence.

In this study, the notion of using the crow search algorithm to estimate the initial solution of the mobile station position in an indoor laboratory setting is proposed. It is also suggested that the iterative crow search method be extended to include the optimization operation known as particle swarm optimization. The TDOA/AOA hybrid positioning is then subjected to the improved crow search algorithm (ICSA) algorithm so that the crow search algorithm can converge quickly, accomplish the optimization goal, and retain its optimization mechanism based on enhancing the performance of the original algorithm. To obtain a better fitness value throughout the optimization procedure and increase the algorithm’s optimization accuracy, the fitness function based on maximum likelihood estimation is modified. To avoid pointless global search and speed up algorithm convergence without compromising population diversity, the initial solution is simultaneously inserted into the starting population location. Simulation findings demonstrate that this approach outperforms TDOA/AOA and other comparable algorithms in terms of node positioning accuracy, convergence speed, and resilience. It also performs better than PSO, CSA, and other algorithms.

## 3. TDOA/AOA Hybrid Location Algorithm

**Figure 1:**
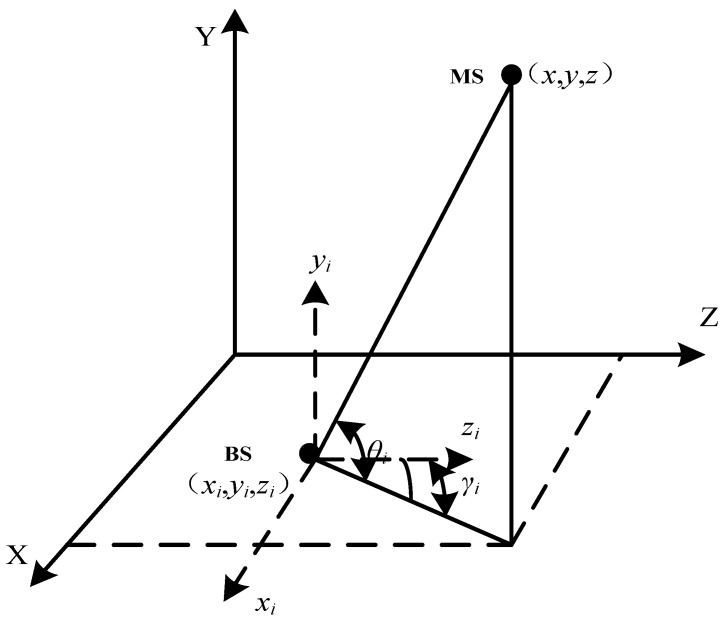
TDOA/AOA joint positioning system model diagram.

The combined usage of the two algorithm models can greatly increase positioning accuracy and lessen the impact caused by measurement mistakes when compared to the TDOA positioning algorithm or the AOA positioning algorithm used alone. To establish a three-dimensional right-angle coordinate system for TDOA/AOA hybrid positioning, in order to simplify the calculation, the base station coordinates are (*x_i_*, *y_i_*, *z_i_*), mobile station MS coordinates (*x*, *y*, *z*), and *z* takes the value of 0, then the base station and mobile station are in the two-dimensional plane. The TDOA/AOA joint positioning system model diagram is shown in Figure 1 [31].

Assuming that *M* base station receivers are arranged in a two-dimensional plane, the position of the *i*th base station *BS* is represented by the coordinates (*x_i_*, *y_i_*), the position of the mobile station *MS* is represented by the coordinates (*x*, *y*), and the distance from the mobile station to the base station is *r_i_*, then the distance equation can be listed according to the (TOA) measurements:

Suppose there are *M* base station receivers arbitrarily distributed in a two-dimensional plane, where the location of the *i*th base station receiver is (*x_i_*, *y_i_*) and the location of the mobile station is (*x*, *y*). The distance from the mobile station to base station *i* is *r_i_*, The difference between the actual distance from the mobile station to base station *i* (*i* ≠ 1) and base station 1 is noted as $r_{i,1}^{0}$, and the measured value is noted as $r_{i,1}^{}$.

The measured value of the time difference of arrival TDOA is converted to the distance difference *r_i,1_*, and *r_i,1_* denotes the distance difference between the mobile station to base station *i* (*i* ≠ 1) and to base station 1, i.e.:

(1) $$r_{i,1}^{}=c\tau_{i,1}=r_{i,1}^{0}+n_{i,1}=r_{i}-r_{1}+n_{i,1}$$

where $\tau_{i,1},i=2,\ldots ,M$ is the TDOA measurement; *c* is the radio wave propagation speed; and $n_{i,1},i=2,\ldots ,M$ is the noise introduced when detecting the TDOA measurement. When the SNR is high, the TDOA measurements detected by Generalized Cross-Correlation (GCC) are usually Gaussian data and obey an approximate normal distribution, so the noise $n_{i,1}$ also obeys an approximate normal distribution, which for convenience can be considered to have a mean value of 0 and a variance of $\sigma^{2}$.

As

(2) $$r_{i}=c\tau_{i}=\sqrt{{\left(x_{i}-x\right)}^{2}+{\left(y_{i}-y\right)}^{2}}$$

therefore, there are:

(3) $$r_{i,1}=r_{i}-r_{1}+n_{i,1}=\sqrt{{\left(x_{i}-x\right)}^{2}+{\left(y_{i}-y\right)}^{2}}-\sqrt{{\left(x_{1}-x\right)}^{2}+{\left(y_{1}-y\right)}^{2}}+n_{i,1}$$

Assuming that the service BS always provides the AOA measurement of the MS, the equation can be established based on the AOA measurement α

(4) $$\alpha =\arctan(\frac{y-Y_{1}}{x-X_{1}})+n_{\alpha }$$

where $n_{\alpha }$ is the AOA measurement error, which follows a normal distribution with mean 0 and variance $std^{2}\alpha$.

Noted as: $\Delta r={\left[r_{2,1},r_{2,1},\ldots ,r_{M,1}\right]}^{T}$, $r={\left[r_{2},r_{3},\ldots ,r_{M}\right]}^{T}$, $r_{1}={\left[r_{1},r_{1},\ldots ,r_{1}\right]}^{T}$, $n={\left[n_{2,1},n_{3,1},\ldots ,n_{M,1}\right]}^{T}$, then there are:

(5) $$\Delta r=r-r_{1}+n$$

when *M* > 3, that is, when the number of valid measurement base stations is 4 or more, in order for the algorithm in this paper to make full use of the statistical information of all the TDOA measurements provided by the network and AOA measurements of the service BS. The maximum likelihood method (ML) is used to determine the mobile station location because $r_{i,1}$ obeys a normal distribution with mean (*r_i_*-*r*_1_) and variance *σ^2^* and *α* obeys a normal distribution with mean $\arctan\left(\frac{y-y_{1}}{x-x_{1}}\right)$ and variance $std^{2}\alpha$, assuming that all measurements are independent of each other, the maximum likelihood estimate of the mobile station location is

(6) $$\left(x,y\right)=\arg\min[{\left(\Delta r-r+r_{1}\right)}^{T}\left(\Delta r-r+r_{1}\right)+\frac{\sigma^{2}}{\alpha^{2}}{\left(\alpha -\arctan\left(\frac{y-y_{1}}{x-x_{1}}\right)\right)}^{2}]$$

In order to obtain the best answer for the coordinate values, a modified crow search algorithm is employed since, in fact, it is exceedingly challenging to solve the minimum of the nonlinear function of Equation (6) using the general technique and results are tough to achieve.

## 4. Crow Search Algorithm

An algorithm inspired by nature, the crow search algorithm (CSA) was developed by Askazadeh (2016) [32]. Crow behavior and social interactions are simulated by this population-based algorithm for evolutionary computational methods. Crows are undoubtedly clever birds since they live in groups, have huge brains compared to their size, and conceal food in areas they can remember and find even months later [33]. In the mirror test, they also demonstrate self-awareness. Through intricate communication, they can retain each other’s facial expressions when hostile crows are around and alert other crows [34]. Crows will occasionally commit thievery by carefully watching the food hiding spots of other crows and then stealing their food, much like other flock animals do. A crow will relocate away from the food cache when it thinks another crow is following it in an attempt to deceive the burglar [35].

Assume that the number of individual crows in the *d*-dimensional optimization problem is *N* and the position of crow *i* at the *t^th^ iteration is* $x^{i,t}=[x_{1}^{i,t},x_{2}^{i,t},\ldots ,x_{d}^{i,t}]$, where *i* = 1, 2, ..., *N*; $t=1,2,\ldots ,t_{\max}$, and $t_{\max}$ is the maximum number of iterations [36]. Each crow has memory and remembers the current optimal food source (i.e., food hiding place). Suppose that the food source of crow *i at the t^th^ iteration is* $m^{i,t}$.

A crow updates its position by observing and following other individuals. Assuming that at the *t^th^* iteration crow *j* flies towards the food source $m^{j,t}$ and crow *i* follows it and approximates $m^{j,t}$ with probability, there will be two scenarios depending on whether crow *j* perceives being followed or not [37].

Scenario 1: Crow *j* does not notice being followed and continues to fly towards $m^{j,t}$, then crow *i* will approach $m^{j,t}$, so its new position at the t+1 iteration is

(7) $$x^{i,t+1}=x^{i,t}+r_{i}\times fl^{i,t}\times (m^{j,t}-x^{i,t})$$

where $r_{i}$ is a random number in the interval [0, 1] and $fl^{i,t}$ is the flight length of crow *i* at the *t^th^* iteration.

Scenario 2: Crow *j* perceives that it is being followed, then it flies randomly in the search space to lure crow *i* to fly to any location in the solution space in order to ensure that the food is not stolen.

The new position of the offspring of crow *i is* known from the above description as

(8) $$x^{i,t+1}=\{\begin{array}{c} x^{i,t}+r_{i}\times fl^{i,t}\times (m^{j,t}-x^{i,t}),r_{j}\geq AP^{j,t}\\ a\;\mathrm{random}\;\mathrm{position},\;\mathrm{otherwise} \end{array}$$

where $AP^{j,t}$ is the perceived probability of being followed at the *t^th^* iteration of crow *j*.

The validity of the solution must be verified after each individual crow position has been updated; if the new position falls within the valid interval, it is updated; otherwise, the parent position is taken. Subsequently, the fitness value (objective function value) f(⋅) of each individual in the offspring crow population is calculated and the memory is updated according to the principle of minimum/maximum optimization:

(9) $$m^{i,t+1}=\{\begin{array}{c} x^{i,t+1},f(x^{i,t+1})\;is\;\mathrm{better}\;\mathrm{than}\;f(m^{i,t})\\ m^{i,t},\mathrm{otherwise}. \end{array}$$

The CSA algorithm controls the global and local search process of the algorithm by two model parameters—perceptual probability *AP* and flight direction length *fl*, using *N* crows to search and finally find the optimal solution of the problem after $t_{\max}$ parallel iterations [38,39].

## 5. Improving the Crow Search Algorithm

### 5.1. Basic Particle Swarm Algorithm

The biological study of birds engaging in foraging behavior served as the basis for the PSO algorithm. Kennedy et al. developed the particle swarm method by researching the cooperative foraging behavior of bird groups [40].

The biggest food supply is the goal of the particle swarm algorithm, and individual birds are considered as inert particles with no living features [41]. The search procedure goes as follows: initially, there are random particles in the space of the ultimate solutions. Individual particles then search for the final solution in the space at a moment’s notice, memorizing the distance closest to the final solution during the search as the current individual extreme value, and then communicating this distance information to other particles in the particle swarm [42]. The overall extreme value of the swarm is the best individual extreme value of the whole swarm. Next, the particles’ individual and collective extremes are used to modify their direction and speed. Eventually, after multiple rounds, the majority of the particles will congregate towards the ultimate solution.

Define the inertia factor as *ω*, taking the value of

(10) $$\omega =\omega_{\max}-\frac{z\left(\omega_{\max}-\omega_{\min}\right)}{T}$$

In Equation (10), *ω* ∈ (*ω*_min_, *ω*_max_) gradually changes from large to small as the particles evolve iteratively. $c_{1}$ with $c_{2}$ are used as the learning factor also known as the acceleration constant, $x_{i}^{t+1}$ as the position of the *i*th particle in the *t*-dimensional solution space, $gb_{}^{t}$ as the global extremum, and $pb_{i}^{t}$ as the individual extremum, the particle position update formula is given in Equation (11), and the velocity update formula is given in Equation (12).

(11) $$v_{i}^{t+1}=\omega v_{i}^{t}+c_{1}r_{1}(pb_{i}^{t}-x_{i}^{t})+c_{2}r_{2}(gb_{}^{t}-x_{i}^{t}),\;i=1,2,\ldots ,n{\;v}_{\min}\leq v_{i}^{t+1}\leq v_{\max}$$

(12) $$x_{i}^{t+1}=x_{i}^{t}+v_{i}^{t+1},x_{\min}\leq x_{i}^{t+1}\leq x_{\max}$$

### 5.2. Crow Search Algorithm Optimized by Particle Swarm Optimization

The classic crow search method suffers from a single population and unequal distribution during the initial iteration, which makes the algorithm susceptible to local optimums and results in low optimization accuracy. Introduced is the aforementioned particle swarm algorithm. Table 1 displays the PSO-optimized crow search algorithm’s pseudo code.

## 6. Application of the PSO-CSA Algorithm on TDOA/AOA

### 6.1. Adaptation Function

The upgraded crow search algorithm’s foundation for determining the search direction is the fitness function. The moving table’s coordinates (*x*, *y*) are those that correspond to the algorithm’s best fitness. With regard to the fitness function,

(13) $$Fitness\left(Y\right)=[{\left(\Delta r-r+r_{1}\right)}^{T}\left(\Delta r-r+r_{1}\right)+\frac{\sigma^{2}}{\alpha^{2}}{\left(\alpha -\arctan\left(\frac{y-y_{1}}{x-x_{1}}\right)\right)}^{2}]$$

The equation determines each particle’s unique ideal location, where a smaller value symbolizes better particle adaptation to Function (14).

(14) $$q_{kp}\left(z\right)=\left\{ \begin{array}{l} q_{kp}\left(z-1\right),fitness\left(y_{kp}\left(z\right)\right)\geq fitness\left(q_{kp}\left(z-1\right)\right)\\ y_{kp}\left(z\right),fitness\left(y_{kp}\left(z\right)\right)<fitness\left(q_{kp}\left(z-1\right)\right). \end{array}\right.$$

The global optimal position of particle k is determined by Equation (13).

(15) $$\begin{array}{l} q_{gk}\left(z\right)\in \{q_{g1}\left(z\right),\cdots ,q_{gs}\left(z\right)|fitness\left(q_{gs}\left(z\right)\right)=\\ \min\left\{fitness\left(q_{g1}\left(z\right)\right),\cdots |fitness\left(q_{gs}\left(z\right)\right)\right\}\} \end{array}$$

Let the coordinate vector of the particle be defined as

(16) $$\psi_{i}={\left(x_{i},y_{i}\right)}^{T}$$

As shown in Equation (16), (*x_i_*, *y_i_*) is the coordinate point of the mobile station to be estimated. Let the coordinates of the mobile station as (*x*, *y*) be within the range constituted by the base station, i.e.:

(17) $$\left\{ \begin{array}{l} x_{\min}\leq x\leq x_{\max}\\ y_{\min}\leq y\leq y_{\max} \end{array}\right.$$

As shown in Equation (17), *x*_min_ and *x*_max_ are the minimum and maximum values of the horizontal coordinates in the range formed by the base station and *y*_min_ and *y*_max_ are the minimum and maximum values of the vertical coordinates in the range formed by the base station.

### 6.2. Improving the Implementation Process of the Crow Optimization Algorithm

The following measures may be taken to enhance how the crow optimization method is implemented:

(1) Initialize each parameter of the algorithm: determine the population size *N*, the maximum number of iterations *T*, the flight length *fl*, the perceptual probability AP and other acceleration factors *c*_1_ and *c*_2_, the maximum inertia factor *ω*_max_, and the minimum inertia factor *ω*_min_, and calculate the inertia factor *ω* according to Equation (10);
(2) Set up the crow memory location and population place;
(3) Determine each person’s fitness value in accordance with the fitness Function (13), and set the individual optimum and the global optimum as *P_best_* and *g_best_*;
(4) Randomly select an individual from the previous generation;
(5) Check to see if the random number that was created is greater than the *AP* for discovery. Person *i* makes the decision to follow person *j* when *r_j_* ≥ *AP*. The inertial velocity, the global ideal solution, and the present optimal solution of individual *j* all affect the velocity of individual *i*. The inertial velocity, the global ideal solution, and the present optimal solution of individual *j* all affect the velocity of individual *i*. If not, the local optimal solution, the global ideal solution, and the inertial velocity of individual *i* make up the velocity of that individual. Equation (18) is used to determine the velocity of individual *i* and Equation (12) is used to determine where individual *i* will be in the subsequent iteration. Both the personal and the overall optimums are updated.
  (18) $$\{\begin{array}{c} v_{i}^{t+1}=\omega v_{i}^{t}+c_{3}r_{3}(pb_{j}^{t}-x_{i}^{t})+c_{2}r_{2}(gb_{}^{t}-x_{i}^{t}),\;r_{j}\geq AP,\\ v_{i}^{t+1}=\omega v_{i}^{t}+c_{1}r_{1}(pb_{i}^{t}-x_{i}^{t})+c_{2}r_{2}(gb_{}^{t}-x_{i}^{t}),\;\mathrm{else}. \end{array}$$
  $x_{\min}$ and $x_{\max}$ are the minimum and maximum positions of the particles, respectively. In the aboce, $c_{3}$ indicates the degree of influence of individual *j* on individual *i* and $r_{3}$ is a random number within [0, 1]. The update rate in Equation (18) is also limited by $v_{\min}$ and $v_{\max}$.

(1) Check whether the algorithm converges and if it does, carry on running the program. Go to step 2 if not;
(2) When *M* iterations have been completed, the iteration is terminated, and the best memory value is produced based on the fitness function’s value. If not, proceed to Step (2) again until the termination condition is met.

**Figure 2:**
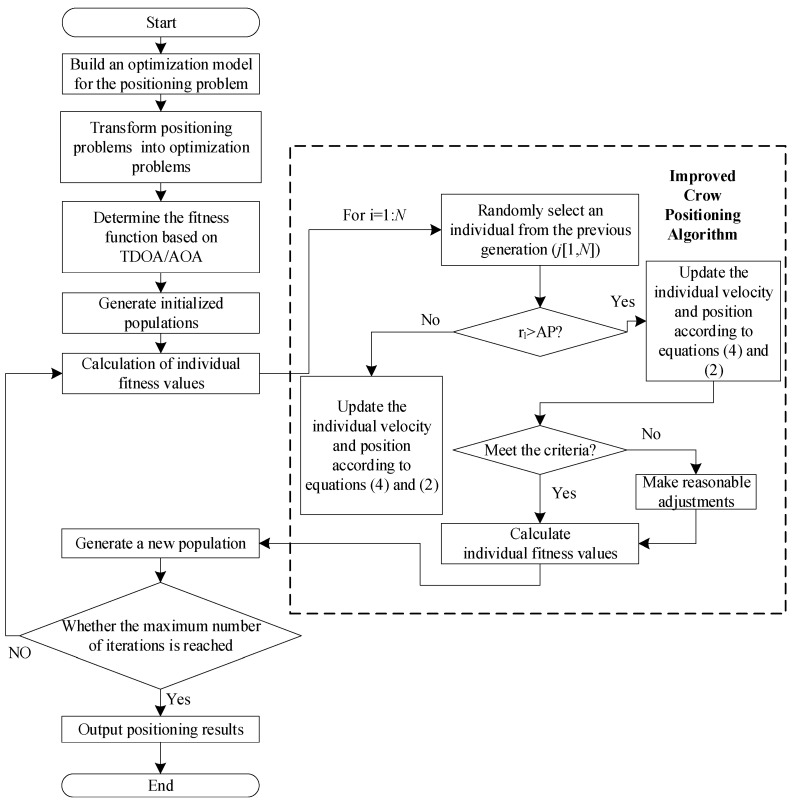
Flow chart the localization algorithm based on the PSO-CSA algorithm.

Figure 2 depicts the flow of the enhanced crow search method localization algorithm.

Figure 2 is the workflow of the mobile position positioning algorithm of the PSO-CSA algorithm. Firstly, the mobile location positioning problem is transformed into a combinatorial optimization problem under constraints, and its minimum positioning error is obtained. The objective function of this TDOA/AOA hybrid positioning algorithm is converted into a fitness function value, and it is solved iteratively through the particle swarm optimization crow search algorithm proposed in this paper. The crow search algorithm is used to complete the search for the optimal solution to more complex spatial problems through the cooperation among the crow populations, and all the crow groups search towards the direction of the individual optimal position and the direction of the global optimal position. However, during the search process, the excellent crow individuals searched in the later period are too concentrated, and the individuals tend to fall into a local optimum. In order to overcome the lack of similar population diversity, this paper introduces the idea of particle swarm optimization to improve population diversity while maintaining the particle concentration within a reasonable range and concludes that the particle swarm optimization crow search algorithm takes the crow search algorithm model as the core, the particle swarm optimization mechanism is introduced to assist in the adjustment of the algorithm.

The PSO algorithm focuses more on optimization efficiency and is closer to the current optimal solution during iteration, resulting in a strong ability to exploit currently known information, while the CSA provides greater freedom for the algorithm to ensure the diversity of solutions, resulting in a stronger ability to explore unknown regions. The proposed PSO-CSA combines the advantages of both PSO algorithms and CSA to achieve a better balance between increasing randomness and improving efficiency.

**Figure 3:**
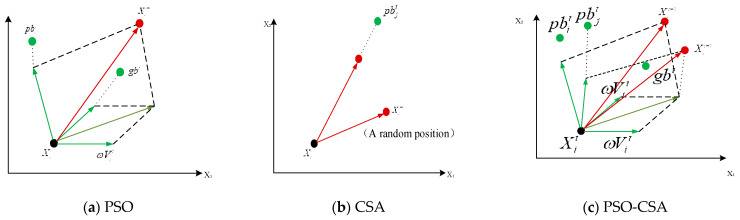
Individual search for optimal solution process of three algorithms.

Presented is a schematic diagram of how the individual in Figure 3 updates their position in (a) PSO, (b) CSA, and (c) PSO-CSA, where the green dots indicate the current best position of the individual, the pink dots indicate the best position determined by the whole population, and the red dots indicate the position of the individual in the next iteration; the red arrows indicate the movement of the individual. The proposed PSO-CSA algorithm combines the advantages of particle swarm optimization and CSA and achieves a better balance between increasing randomness and improving efficiency. The proposed PSO-CSA algorithm increases the iterative process of the algorithm to solve the optimal solution, the diversity of the population, and the calculation speed.

## 7. Algorithm Comparison and Result Analysis

### 7.1. Function Optimization

This work performs comparison tests using six common benchmark functions that represent various challenges in the actual search space in order to evaluate the four population intelligent optimization techniques that are proposed. Table 2 displays the functions’ specifics. For more plausibility, 200 separate experiments were conducted for each test function in all circumstances, with a maximum of 50 iterations and a population size of 50. As demonstrated in Table 3, the precise parameters used in the original study are the parameters chosen by various methods.

**Figure 4:**
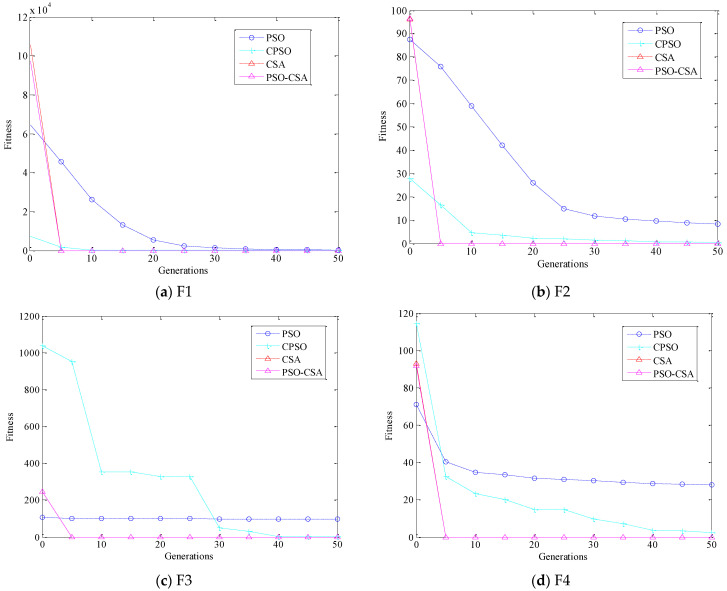

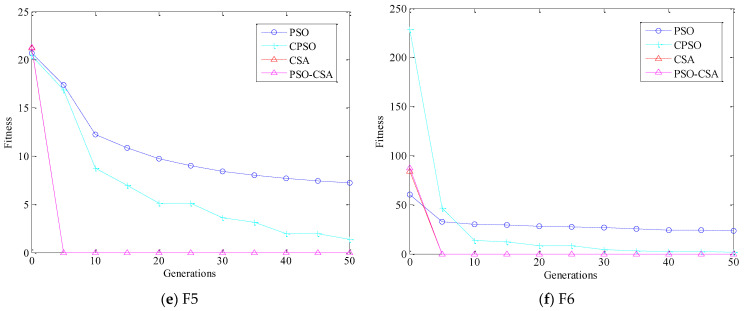
Comparison of iterative calculation results of functions.

The iterative calculation results of the test functions F1–F6 used in this paper are shown in Figure 4a–f. Table 4 presents the experimental results of the four algorithms, PSO-CSA, CSA, CPSO, and PSO, after running 200 times independently on multiple test functions.

A comparison chart of the convergence of the four algorithms on the test function is given in Figure 3 in order to confirm the stability and convergence of the four intelligent optimization methods. When dealing with the test function, the PSO, CPSO, CSA, and PSO-CSA algorithms all exhibit the phenomena of premature convergence. Even though the crow search method prematurely converges in a few test functions, it nevertheless achieves higher solution accuracy than other algorithms. The accuracy of the answer at this point is higher than that of other methods when the PSO algorithm solves the test functions F1, F2, F3, and F4. Testing functions F5 and F6 improves the performance of the crow search algorithm, and as the number of iterations rises, so does the correctness of the answer. When solving functions F5 and F6, the particle swarm optimization crow search technique presented in this study yields the global best solution in roughly 15 iterations. When compared with other methods, the solution accuracy on other functions is higher and maintains a better ratio of local development to global search. In general, CSA and PSO-CSA have greater optimization capabilities than other algorithms and PSO-CSA’s effect is superior than CSA’s. PSO has a mediocre solution impact, whereas CSA has a moderate optimization effect.

### 7.2. Comparison of Positioning Simulation Experiments

#### 7.2.1. Experimental Scenarios and Evaluation Metrics

The algorithm’s experimental setting is built on a Windows 10 64-bit PC running the MATLAB2018b platform. The performance of the Taylor method, Chan algorithm, TDOA/AOA hybrid algorithm, chaotic CSA algorithm, and PSO CSA algorithm is compared in this work. The chosen base stations are greater than three since three base stations operate poorly. The following are the primary criteria used in this study: Use the nine-receiver cellular layout and select between four and nine base stations. The serving base station is BS1, and the cell radius has been set at 3000 m. The base station’s coordinates are chosen as follows: BS1 (0,0), BS2 ($-\sqrt{3}$, 0), BS3 ($\sqrt{3}$, 0), BS4 ($\sqrt{3}$/2, 3/2), BS5 ($-\sqrt{3}$/2, −3/2), BS6 ($-\sqrt{3}$/2, 3/2), BS7 ($\sqrt{3}$/2, $-\sqrt{3}$/2), BS8 (0, 2), BS9 (0, −2). A Gaussian normal distribution with a mean value of 0 and variances of 30, 60, 90, 120, and 150 m, respectively, governs the measurement error via TDOA. The channel model is satisfied by the non-line-of-sight error brought on by the channel environment, and the serving BS’s AOA measurement error follows a Gaussian normal distribution with a mean of 0. The starting inertia value max of the PSO algorithm is 0.9, the inertia weight min while iterating to the optimum algebra is 0.2, the initial number of particles is 60, and the iteration count is 20. The learning factors *c*_1_ and *c*_2_ of the PSO method are 2.4. The CSA algorithm’s attentiveness probability (AP) is 0.1 and the flight length (*fl*) is 2.

The environment-related parameters in wireless positioning are shown in Table 5.

#### 7.2.2. Number of Base Stations, Cell Radius, and Measurement Error as Variables to Compare Algorithm Positioning Performance

**Figure 5:**
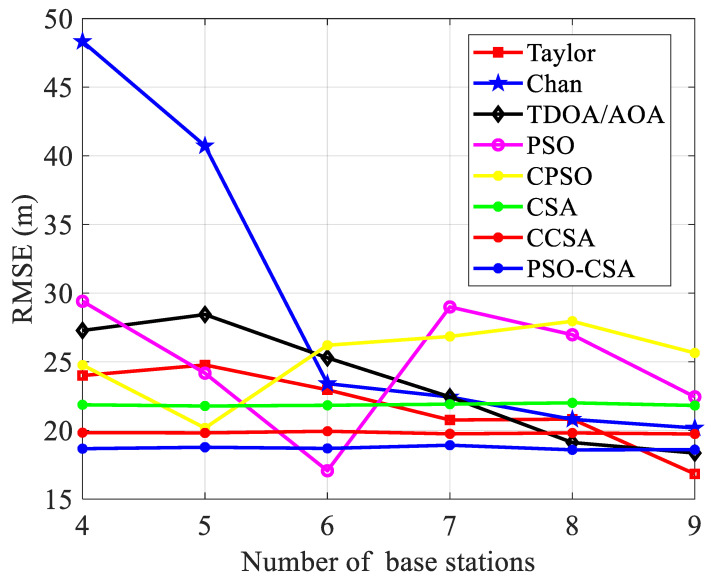
Comparison between standard error and base station.

(1) The quantity of base stations has an impact on the positioning performance. Figure 5 shows that the mobile station’s beginning coordinates are set to (0.8, 0.2). and that the inaccuracy is 30 m, the radius is 3000 m. From 4 to 9, there are nine base stations, and each algorithm’s location accuracy keeps improving as the standard error becomes smaller. The positioning efficiency of the PSO CSA algorithm is the best overall, followed by the CCSA algorithm and the classic CSA, and the other methods are organized in turn. Overall, the curve of the PSO CSA algorithm is substantially smaller compared to the other algorithms.

The positioning accuracy of the PSO-CSA method and its enhanced algorithm have greatly outperformed the Taylor algorithm, Chan algorithm, and TDOA/AOA algorithm, as can be seen from the overall average in Figure 5. The CSA method and its upgraded algorithm exhibit smooth curves from the perspective of stability, which demonstrates the greater stability of the algorithm.

**Figure 6:**
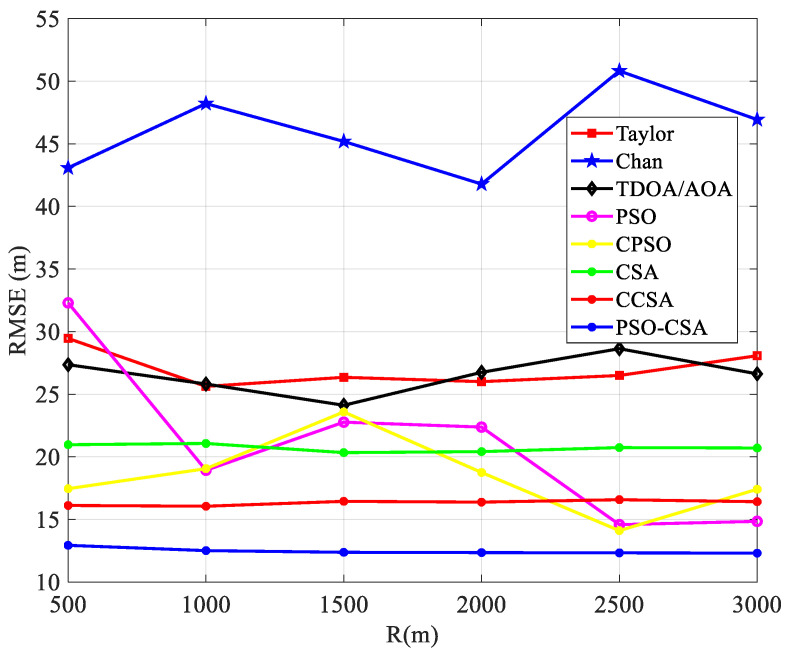
Comparison between quasi error and cell radius.

(2) The cell radius has an impact on positioning performance. As observed in Figure 6, the positioning error exhibits an increased trend as the cell radius continues to grow in the scenario with four base stations and a measurement inaccuracy of 30 m. Figure 6 depicts the link between standard error and cell radius.

Figure 6 illustrates how the PSO-CSA method outperforms other positioning algorithms in terms of positioning performance and reliability. This is because the PSO-CSA algorithm optimizes the TDOA/AOA functional formula, which removes certain mistakes, substantially lowers errors brought on by radius changes, and increases the precision of positioning.

**Figure 7:**
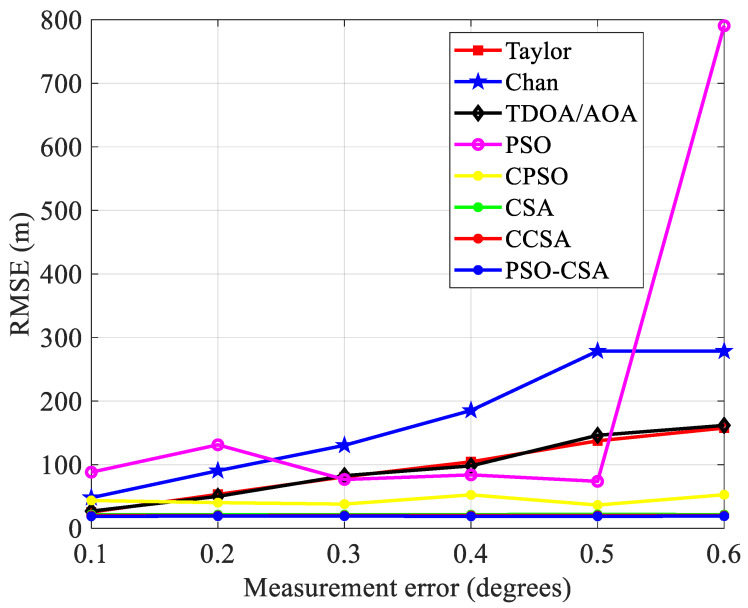
The correlation between the measurement error and the base station’s four standard errors.

**Figure 8:**
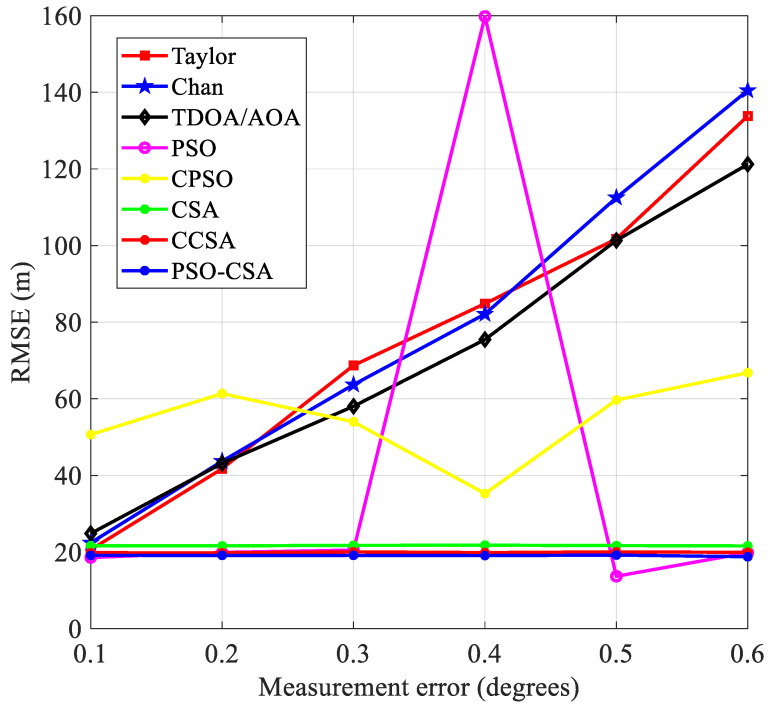
Comparison of standard error and measurement error.

(3) The measurement inaccuracy has an impact on positioning performance. As observed in Figure 7, The measurement error *x* = *σ_AOA_* × *c*, where the parameter *c* is the speed of light, when the radius is 3000 m and the base station is 7. The measurement error variance is 30 m to 240 m. The standard error grows in proportion to the measurement inaccuracy. Figure 8 depicts the connection between the measurement error and the standard error.

In Figure 8, the CSA method and its upgraded algorithm have stronger stability and greatly improved positioning performance when compared with other algorithms. Errors have a significant impact on alternative placement techniques. The chance of divergence in the final measurement result is higher and the algorithm’s performance is more unstable as the measurement error rises.

Four base stations are selected under the same other parameters and Figure 7 depicts their relationship.

The standard error has grown relative to the mean, indicating that the number of base stations has a significant impact on the root mean square error. The TDOA and AOA readings will be erroneous due to the decrease in base stations, increasing inaccuracy.

**Figure 9:**
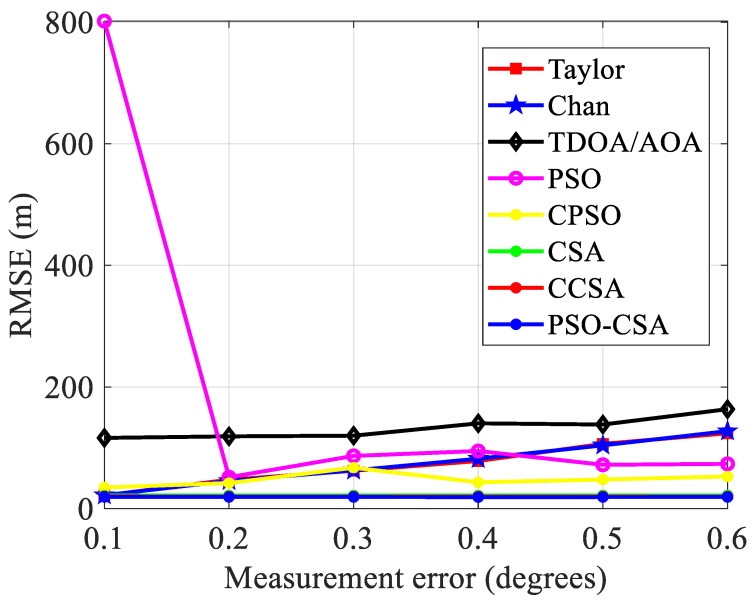
The correlation between measurement error and standard error at various datum locations.

Figure 9 depicts the link between the reference coordinates (0.8, 0.6) and the selection of seven base stations.

As observed in Figure 9, the CSA algorithm and its upgraded algorithm work better and achieve superior positioning accuracy when they are near the base station’s center. As a result, the reference coordinates used throughout the measurement procedure can improve the benefits of the positioning algorithm. However, the traditional CSA method and the enhanced algorithm are not that dissimilar.

(4) The correlation between mean square error, cell radius, and number of base stations. Using Formula (19) and 200 experiments, the position estimate *MSE* is determined as follows: *y* = 10lg (*MSE*). Let the width of the abscissa be the amount of measurement error, the number of base stations, and the cell’s radius.

(19) $$MSE=\frac{{\sum_{L=1}^{200}\left\|\tilde{x}\left(l\right)-x\right\|}_{}^{2}}{200}$$

**Figure 10:**
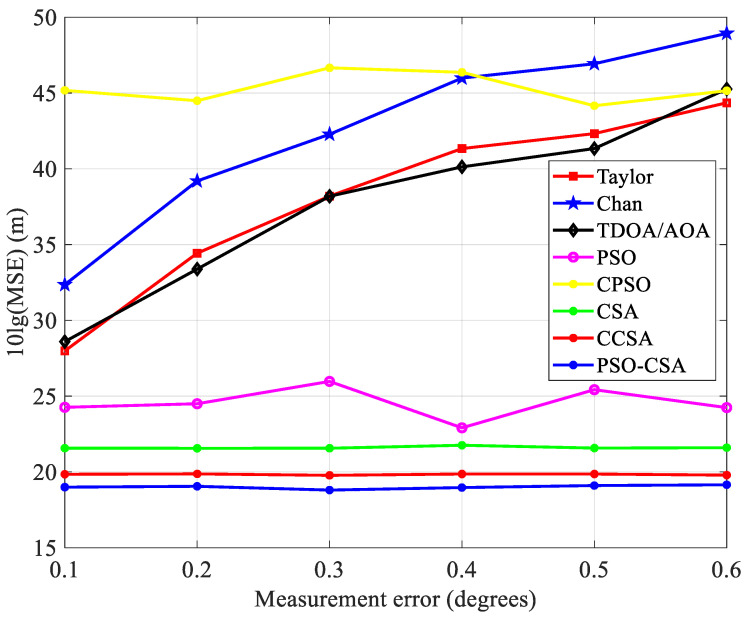
Mean squared error and measurement error comparison.

**Figure 11:**
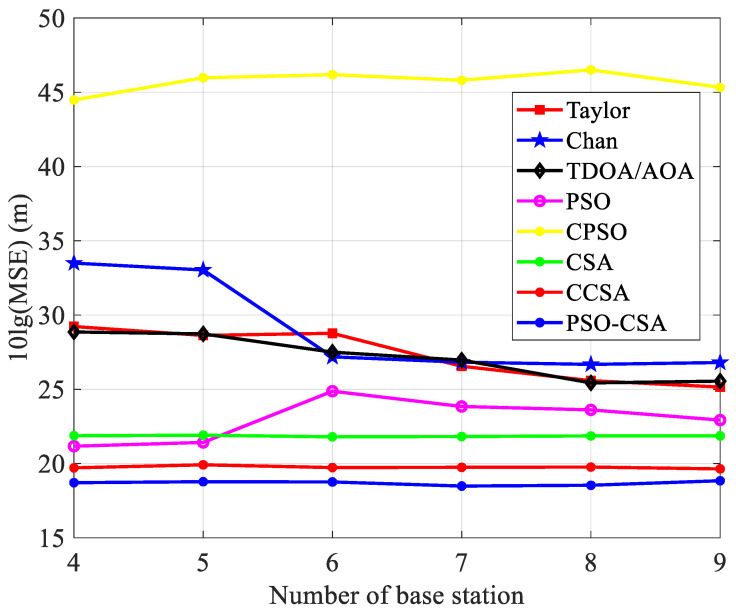
Base station counts and mean square error comparison.

**Figure 12:**
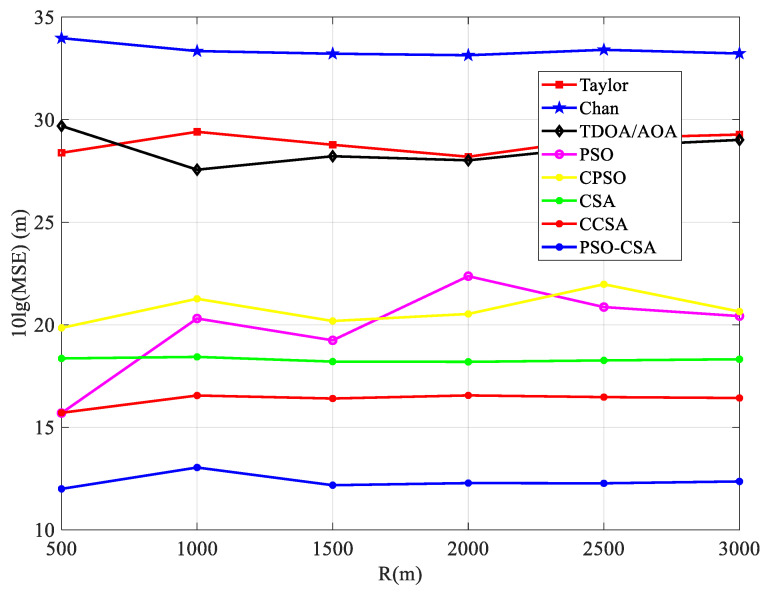
Radius and mean squared error comparison.

As shown in Equation (19), $\tilde{x}\left(l\right)$ is the estimated position value of the *l*-th time of equation *x*. The measurement results are shown in Figure 10, Figure 11 and Figure 12.

Figure 10 illustrates this relationship between measurement error and mean square error. The PSO-CSA method exhibits good positioning performance in the mean square error. This is because the PSO-CSA algorithm improves positioning accuracy by minimizing the impact of measurement error on placement. Compared with the Taylor algorithm, Chan algorithm, TDOA/AOA method, PSO algorithm, and CPSO algorithm, the CSA algorithm and its upgraded algorithm have smoother curves and more stable effects.

The placement accuracy of the PSO algorithm is marginally superior to that of the CSA method when there are four to five base stations, as illustrated in Figure 11. The PSO-CSA method presented in this study has much greater placement accuracy than existing algorithms. The accuracy is not significantly impacted by the number of base stations, and the CSA algorithm performs better than the PSO method when there are more than five base stations. As the measurement value of the TDOA between the BS and MS has been adjusted, the positioning performance is now affected by the measurement variance of the AOA.

According to Figure 12, the positioning performance of the PSO-CSA algorithm proposed in this paper is superior to that of other algorithms. This is because the algorithm uses ergodicity to avoid nonlinear linearization during operation, which causes a problem with the algorithm settling for the local optimal solution. The PSO-CSA method, which can effectively solve the nonlinear issue and enhance the positioning accuracy of the algorithm, is employed in TDOA/AOA hybrid positioning since the positioning performance of TDOA/AOA hybrid positioning has been enhanced.

(5) The eight methods’ 3D positioning error results

**Figure 13:**
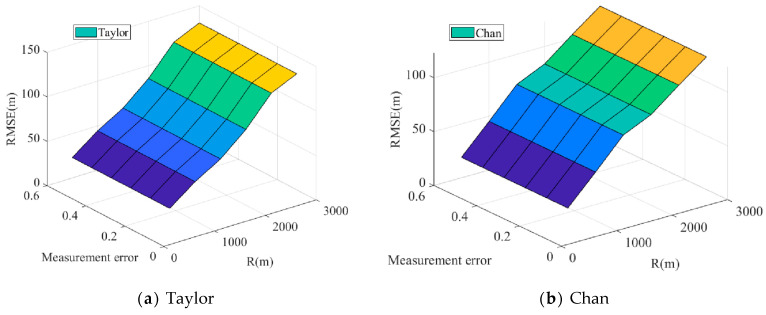

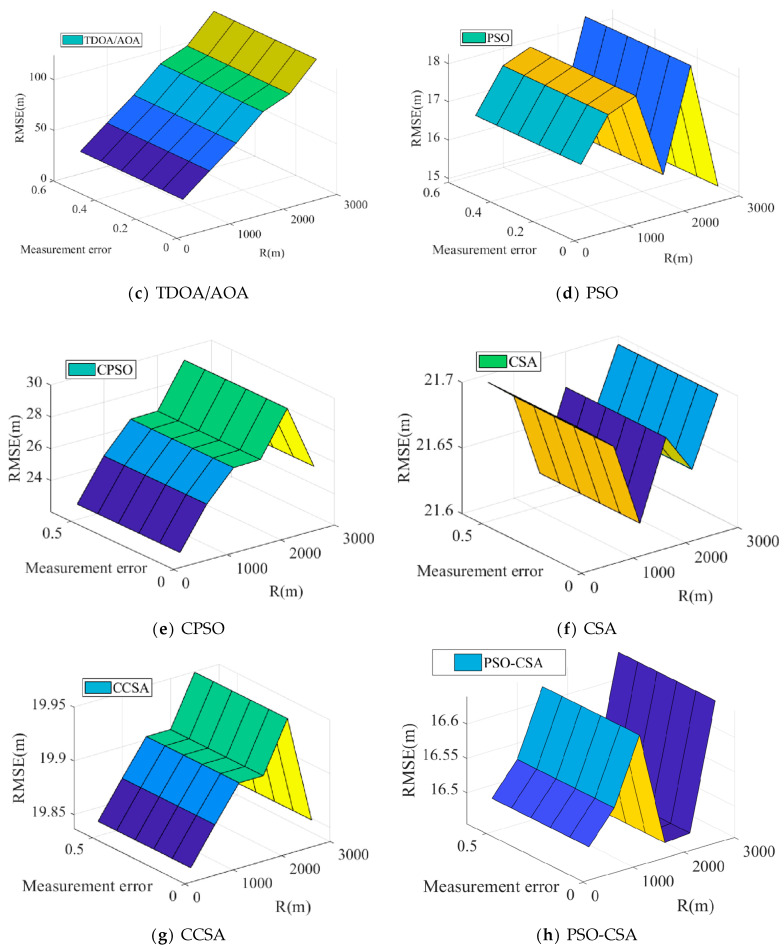
Comparison of 3D positioning errors.

This study examines the positioning capabilities of the Taylor algorithm, Chan algorithm, TDOA/AOA hybrid algorithm, PSO algorithm, CPSO method, CSA algorithm, and our modified CSA algorithm in terms of three-dimensional space placement on the basis of two-dimensional space. Figure 13a–h illustrates the results of the 3D localization algorithm, accordingly.

The root mean square error of the Taylor algorithm, Chan algorithm, TDOA/AOA hybrid algorithm, PSO algorithm, CPSO algorithm, CSA algorithm, and other algorithms will increase with an increase in measurement error and communication radius, it can be seen from the comparison of the three-dimensional positioning error of these eight algorithms. Among these, the Taylor algorithm’s maximum positioning error is 130.5 m, the Chan algorithm’s is 122.9 m, and the TDOA/AOA hybrid algorithm’s is 123.9 m. The PSO method for swarm intelligence optimization has a positioning error of 101.8 m, whereas the CPSO algorithm has a positioning error of 29.23 m. The SSA algorithm’s positioning error is 21.69 m, whereas the CCSA algorithm’s positioning error is 19.95 m. The positioning effects of the CSA algorithm and the CCSA algorithm are better than those of the PSO algorithm and the CCSA algorithm, and it can be shown that the mistakes of the swarm intelligence optimization algorithm are fewer than those of the conventional three positioning techniques. In comparison with previous positioning algorithms, the PSO-CSA technique introduced in this research has a positioning error of 16.64 m. The PSO-CSA method suggested in this research offers the best positioning impact and the minimum positioning error, as can be observed.

(6) Comparison of time required

**Figure 14:**
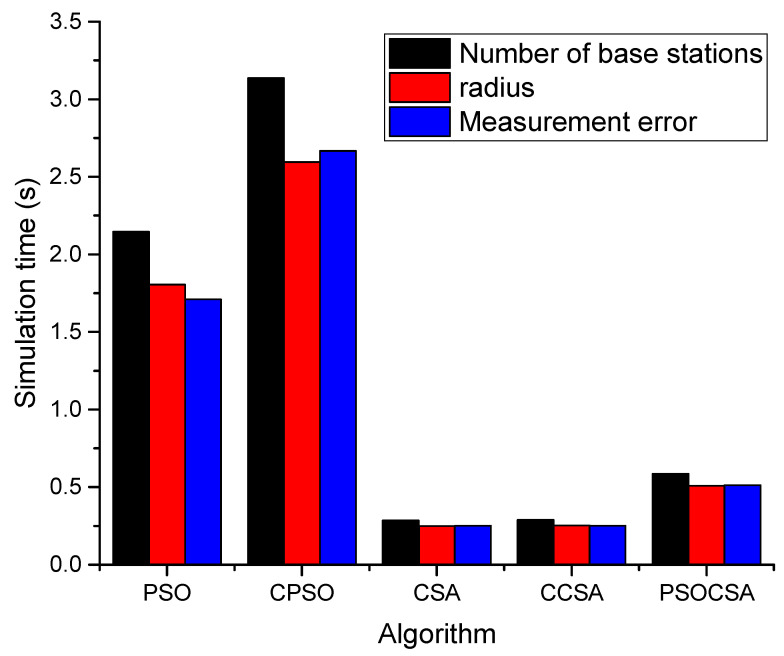
Comparison of algorithm in terms of time requirement.

We compared the simulation time of five algorithms including PSO, CPSO, CSA, CCSA algorithm and PSOCSA, as shown in Figure 14. It can be seen that the CSA and CCSA algorithms and their improved algorithms are less time consuming, followed by the PSOCSA algorithm proposed in this paper. The main particle swarm optimization algorithm takes some time to optimize and solve the overshoot. The PSO algorithm and the CPSO algorithm take more time, mainly because the chaos algorithm needs to be optimized first, and the simulation needs more time than the PSO algorithm. Compared with the CSA algorithm and the CCSA algorithm, the PSO-CSA algorithm takes less time.

## 8. Conclusions

In this work, we examine how the PSO-CSA method may be used to improve the TDOA/AOA hybrid localization technique in an indoor laboratory setting utilizing particle swarm optimization. The problem of TDOA/AOA hybrid positioning, which is significantly impacted by errors and nonlinear optimization, is successfully resolved by using the function of TDOA/AOA hybrid positioning as a fitness function for optimization and finding the coordinate point corresponding to the optimum fitness. The simulation results demonstrate that the proposed PSO-CSA method outperforms existing algorithms of the same kind when compared with TDOA/AOA, PSO, CPSO, and CSA algorithms. The program also performs well in terms of node positioning accuracy, convergence speed, and resilience. The chaotic PSO algorithm’s TDOA/AOA hybrid localization technique has research importance in real-world applications because of its simplicity.

With the most recent swarm intelligence bionic optimization algorithm, we will soon enhance the positioning algorithm in both indoor laboratory environments and outdoor environments in order to increase the positioning accuracy of the algorithm.

## Figures and Tables

**Table 1:** The pseudo code of the CSA algorithm optimized by PSO.

Step 1: Establish the size of the swarm *N* with dimension (*D*) same as the number dataset’s attributes.
Step 2: Obtain the *c*_1_, *c*_2_, the weight factor are *w*_max_ and *w*_min_, the maximum velocity is *v*_max_, the flight length is *fl*, the awareness probability is *AP*, and the maximum iteration is *max_iter*.
Step 3: The population is randomly set as *q_i_*,*t* for each solution and *D* dimensional vector as the velocity.
Step 4: set *t* = 0.
$\mathrm{Step}\;5:\;\omega =\omega_{\max}-\frac{z\left(\omega_{\max}-\omega_{\min}\right)}{T}$.
Step 6: The fitness value is set for each of the solution as evaluated function while *P_best_* and *g_best_* values are set.
Step 7: Run CSA with *q_i_*, *t* as the population, a set of crows with the best foods to be followed and a minimum crow.
Step 8: Inversely mutate the returned position by the CSA.
Step 9: Update the position of the swarms.
Step 10: for *k* = 1 to *SS*
$\mathrm{Step}\;11:\;v_{i}^{t+1}=\omega v_{i}^{t}+c_{1}r_{1}(pb_{i}^{t}-x_{i}^{t})+c_{2}r_{2}(gb_{}^{t}-x_{i}^{t}),\;i=1,2,\ldots ,n{\;v}_{\min}\leq v_{i}^{t+1}\leq v_{\max}$
Step 12: end
Step 13: for *k* = 1 to *SS*
Step 14: for *j* = 1 to *D*
Step 15: if (*v*(*i*,*j*) greater than *V*_max_)
Step 16: *v*(*i*,*j*) equal to *V*_max_
Step 17: end
Step 18: if (*v*(*i*,*j*) less than–*V*_max_)
Step 19: *v* (*i*,*j*) equal to–*V*_max_
Step 20: end
Step 21: if (*rand* less than *s*)
Step 22: *q_i_*, *j*, *t* + 1 equal to 1
Step 23: else
Step 24: *q_i_*, *j*, *t* + 1 equal to 0
Step 25: end
Step 26: Produce the best solution

**Table 2:** Test functions.

Function	Equation	Dimension	Bounds	Optimum
F1	$\sum_{i=1}^{d}\overset{2}{\underset{i}{x}}$	30	[−100, 100]	0
F2	$\sum_{i=1}^{d}{\left(\left|x_{i}+0.5\right|\right)}^{2}$	30	[−100, 100]	0
F3	$-20\exp\left(-0.2\sqrt{\frac{1}{d}\sum_{i=1}^{d}x^{2}}\right)-\exp\left(\frac{1}{d}\sum_{i=1}^{d}\cos(2\pi x_{i})\right)+20+\exp(1)$	30	[−5.12, 5.12]	0
F4	${\left(\frac{1}{500}+\sum_{j=1}^{25}\frac{1}{j+\sum_{i=1}^{2}{\left(x_{i}-a_{ij}\right)}^{6}}\right)}^{-1}$	30	[−65, 65]	0
F5	$-\sum_{i=1}^{5}{\left[(X-a_{i}){\left(X-a_{i}\right)}^{T}+c_{i}\right]}^{-1}$	30	[0, 10]	−10.15
F6	$-\sum_{i=1}^{4}c_{i}\exp\left(-\sum_{j=1}^{6}a_{ij}{\left(x_{j}-p_{ij}\right)}^{2}\right)$	30	[0, 1]	−3.32

**Table 3:** Settings of algorithm control parameters.

Algorithm	Parameter	Value
PSO	Learning factor (*C*_1_, *C*_2_)	2
	Inertia weighting factor (*w*_1_, *w*_2_)	0.9, 0.4
CPSO	Learning factor (*C*_1_, *C*_2_)	2
	Inertia weighting factor (*w*_1_, *w*_2_)	0.9, 0.4
CSA	Number of discoverers	20%
	Number of dangerous sparrows predicted	10%
	Safety threshold	0.8
CCSA	The proportion of discoverers (PD)	20%
	Proportion of investigators (SD)	10%
	Warning value (ST)	0.8
PSO-CSA	The proportion of discoverers (PD)	20%
	Proportion of investigators (SD)	10%
	Warning value (ST)	0.8
	*C*_1_, *C*_2_	2, 2
	*w*_1_, *w*_2_	0.9, 0.4
	*V*_max_	6

**Table 4:** Experimental results of function testing.

Function	PSO-CSA			CSA			CPSO			PSO		
	Max	Mean	Std	Max	Mean	Std	Max	Mean	Std	Max	Mean	Std
F1	4.68 × 10^2^	3.21 × 10^2^	2.23 × 10	4.89 × 10^2^	5.24 × 10	6.42	3.04 × 10	5.17	1.24 × 10	1.27 × 10	1.35 × 10	1.58 × 10
F2	3.18 × 10	3.47 × 10^2^	6.17	1.07 × 10^3^	5.84 × 10^2^	8.51 × 10	1.22 × 10^3^	5.45 × 10^2^	3.21 × 10^2^	2.95 × 10	2.34 × 10	1.84 × 10
F3	1.81	3.87	4.15	2.21	3.47	4.62 × 10^-1^	2.34 × 10	4.87 × 10	2.89 × 10	2.45	2.22 × 10^2^	3.14 × 10^2^
F4	2.98	5.12	1.74	1.23 × 10	1.16 × 10	1.35 × 10^-1^	4.04	5.86	1.48	3.09	6.97	3.04
F5	1.73 × 10^2^	1.54 × 10^2^	1.81 × 10	1.97 × 10^2^	2.46 × 10^2^	1.54 × 10^-1^	1.21 × 10^2^	1.53 × 10^2^	1.19 × 10	1.54 × 10^2^	2.14 × 10^2^	6.11 × 10
F6	3.67	3.82	2.42 × 10^-1^	1.74 × 10	1.91 × 10	2.14 × 10^-3^	3.48	3.57	2.98 × 10^-1^	4.07	3.12	2.87 × 10^-1^

**Table 5:** Simulation environment parameters.

Name	Values
Number of base stations	4~9
Cell radius	3000 m
Number of initial particles	60
Number of iterations	20
PSO: *c*_1_, *c*_2_	2.4, 2.4
PSO: *ω*_max_, *ω*_min_	0.9, 0.2
CSA: *AP*	0.1
CSA: *fl*	2

## Data Availability

The data used to support the findings of this study are available from the corresponding author upon request.
